# Bio-assisted synthesized Ag(0) nanoparticles stabilized on hybrid of sepiolite and chitin: efficient catalytic system for xanthene synthesis

**DOI:** 10.1038/s41598-020-71866-2

**Published:** 2020-09-17

**Authors:** Fatemeh Ghoreyshi Kahangi, Morteza Mehrdad, Majid M. Heravi, Samahe Sadjadi

**Affiliations:** 1 Department of Chemistry, University Campus 2, University of Guilan, Rasht, 4199613776 Iran; 2 Department of Chemistry, School of Science, Alzahra University, PO Box 1993891176, Vanak, Tehran, Iran; 3 Gas Conversion Department, Faculty of Petrochemicals, Iran Polymer and Petrochemical Institute, PO Box 14975-112, Tehran, Iran

**Keywords:** Chemistry, Catalysis

## Abstract

In this work, with the use of two natural compounds, chitin and sepiolite clay, a novel covalent hybrid is fabricated and applied as a support for the stabilization of silver nanoparticles with the aid of Kombucha extract as a natural reducing agent. The resultant catalytic system, Ag@Sep-N–CH, was characterized via XRD, TEM, FTIR, ICP, SEM, TGA, UV–Vis and BET. It was found that fine Ag(0) nanoparticles with mean diameter of 6.1 ± 1.8 nm were formed on the support and the specific surface area of the catalyst was 130 m^2^ g^−1^. The study of the catalytic performance of Ag@Sep-N–CH for catalyzing synthesis of xanthenes in aqueous media under mild reaction condition confirmed that Ag@Sep-N–CH exhibited high catalytic activity and could promote the reaction of various substrates to furnish the corresponding products in high yields. Moreover, the contribution of both chitin and sepiolite to the catalysis has been affirmed. It was found that hybridization of these two components led to synergistic effects and consequently improved the observed catalytic activity. Notably, the catalyst was recyclable up to several reaction runs.

## Introduction

Use of metallic nanoparticles in catalysis is a hot topic in chemistry and in recent decades various nanocatalysts have been devised for vast chemical transformations. One of the most reported metallic nanoparticles is silver nanoparticle that has the capability of catalyzing numerous reactions. Moreover, this nanoparticle is a promising choice for other applications such as environmental remediation and sensing^1–4^. To form silver nanoparticles, silver salts are reduced via a reducing agent. Classically, chemical reducing reagents such as hydrazine hydrate are used for this purpose^4,5^. In a more eco-friendly approach, bio-based reducing agents such as plant extracts that are cost effective and non-hazardous reducing agents can replace the conventional chemical ones^6^.

Use of natural clays as low cost and available catalyst support is not a new topic and to date various clays have been reported for this purpose. The main advantages of clays are their high thermal and chemical stability and the presence of some functionalities that makes their tuning possible. Moreover, the chemical compositions and morphologies of clays can impart interesting features to them. Sepiolite (Sep) is a natural rod-like clay with compositional formula of Mg_8_Si_12_O_30_(OH)_4_(OH_2_)_4_·nH_2_O^7^. The structural unit in this hydrated Mg–Al silicate^8^ is composed of two tetrahedral silica sheets and a central octahedral sheet containing magnesium^9^. This feature along with the porosity of this clay gave rise to its extensive use for the catalytic applications^10–12^.

As xanthenes exhibit biological and pharmacological properties^13–17^, their synthesis has attained significant attention and numerous reports have been published on the synthesis of diverse derivatives of these heterocycles^18,19^. In fact, xanthenes not only found many applications in chemistry and pharmacology, they have been extensively utilized in other domains such as pH-sensitive fluorescent materials and dyes. As xanthene synthesis is a catalytic process, the key issue is evolving an effective catalyst that benefits from some features such as high activity, low-cost and reusability. Although various catalysts ranging from ionic liquids to nanoparticles have yet been developed, there are some unaddressed shortcomings and challenges^20–24^.

**Figure 1:**
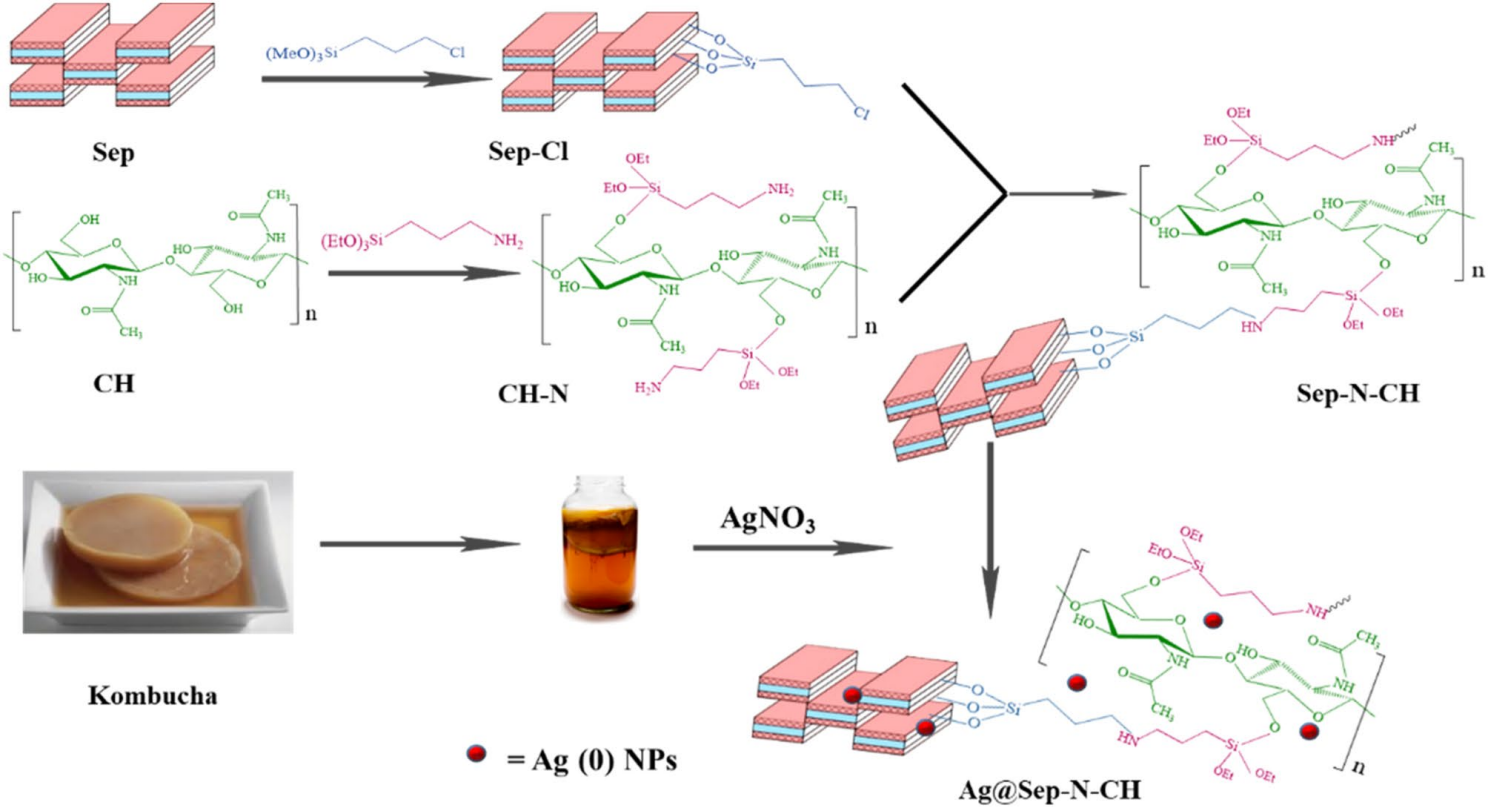
The schematic procedure for the synthesis of Ag@Sep-N–CH.

In the continuation of research on heterogeneous catalysts based on clays and clay-carbohydrate hybrids^25–29^, herein we wish to report a covalent hybrid of sepiolite clay and chitin (CH), Fig. 1, and its utility as a support for the stabilization of silver nanoparticles with assistance of Kombucha extract as a natural reducing agent. The catalytic activity of the resulting hybrid, Ag@Sep-N–CH, was appraised for one-pot synthesis of xanthenes in aqueous media and under mild reaction condition. Furthermore, the roles of sepiolite and chitin in catalysis were evaluated and the recyclability of the catalyst has been examined.

## Characterization

**Figure 2:**
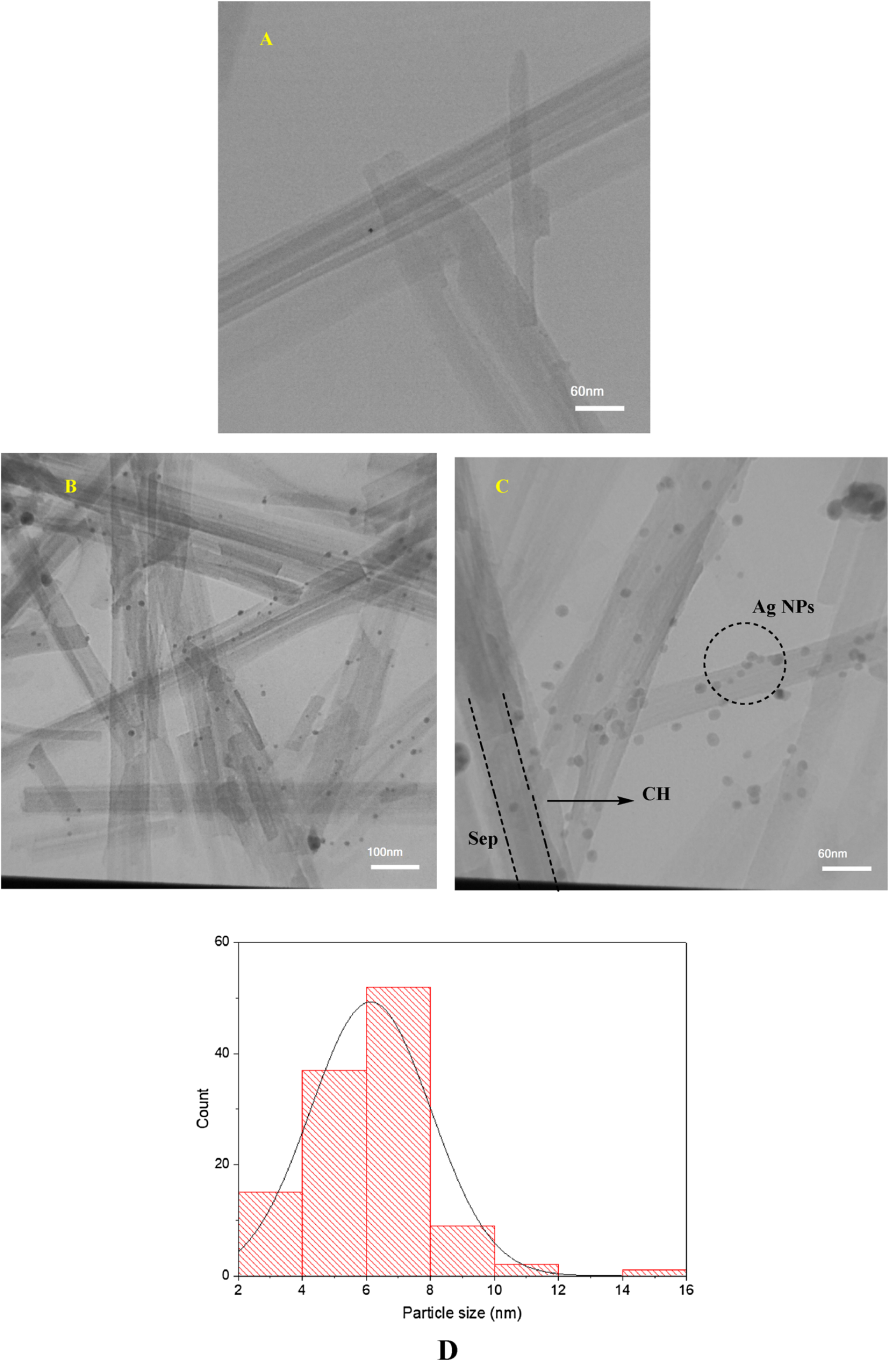
(**A**) TEM image of sepiolite and (**B**, **C**) TEM images of Ag@Sep-N–CH. (**D**) Particle size distribution graph of Ag(0) nanoparticles.

To assess the morphological change of sepiolite after incorporation of CH and Ag(0) nanoparticles, the TEM image of sepiolite (Fig. 2A) and Ag@Sep-N–CH (Fig. 2B,C) were recorded. As shown in Fig. 2A, the TEM analysis of sepiolite revealed that this clay showed rod like morphology. In Ag@Sep-N–CH TEM images (Fig. 2B,C), the sepiolite rods can be discerned, indicating that upon covalent grafting with CH and immobilization of silver nanoparticle, sepiolite morphology did not alter. On the other hand, in some parts of sepiolite rods, fine sheet can be observed that can be ascribed to CH. Furthermore, the spherical black spots on the sepiolite rods are ascribed to the silver nanoparticles. Measurement of silver nanoparticle sizes, Fig. 2D, showed that the mean diameter of these particles was 6.1 ± 1.8 nm.

**Figure 3:**
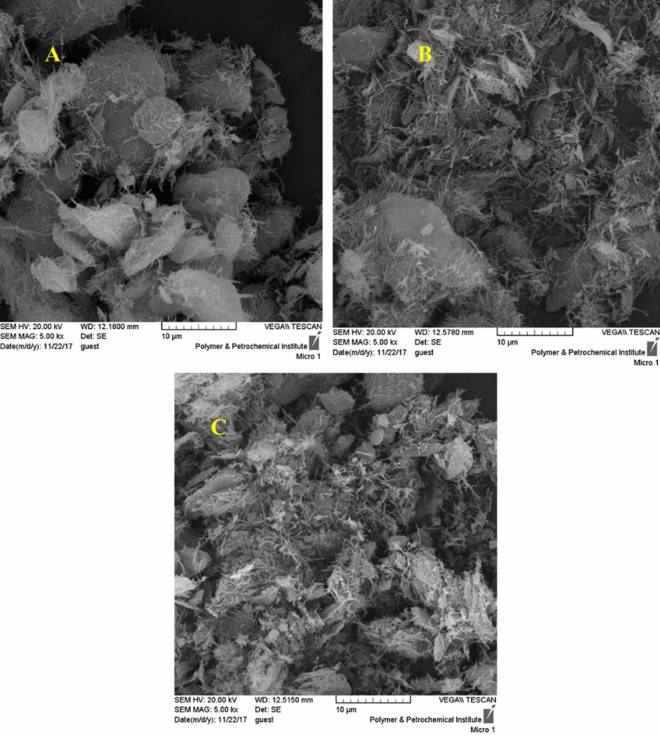
SEM images of (**A**) sepiolite, (**B**) Sep-N–CH and (**C**) Ag@Sep-N–CH.

In Fig. 3, the SEM images of sepiolite (Fig. 3A), Sep-N–CH (Fig. 3B) and Ag@Sep-N–CH (Fig. 3C) are illustrated. As shown, the SEM image of sepiolite is distinguished from Sep-N–CH. This observation indicated that introduction of CH induced morphological change to some extent. The morphology of Ag@Sep-N–CH is almost similar to that of Sep-N–CH, implying that immobilization of silver nanoparticles has slight effect on the morphology. This issue can be due to the low content of silver nanoparticles in the structure of the catalyst.

**Figure 4:**
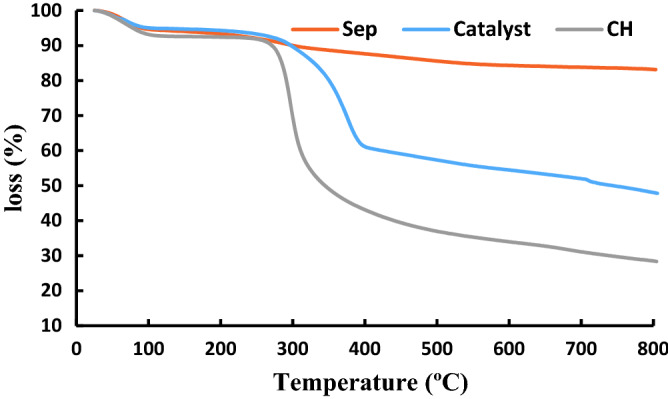
TG curves of CH, Sep and Ag@Sep-N–CH.

To confirm grafting of CH to sepiolite and formation of Ag@Sep-N–CH, TG curve of Ag@Sep-N–CH was compared with that of CH and sepiolite, Fig. 4. As illustrated, among three TG curves shown in Fig. 4, CH and sepiolite showed the lowest and the highest thermal stability respectively. This is a rational result as sepiolite is an inorganic clay with instinctive high thermal stability, while CH is a carbohydrate with low thermal resistance. The thermal stability of Ag@Sep-N–CH is between the two aforementioned samples, implying the conjugation of low thermally stable CH on sepiolite. In more detail, in the TG curve of Ag@Sep-N–CH, the first weight loss, discerned below 200 °C is due to the loss of structural water, while the second one at 380 °C is the sign of degradation of CH. According to TGA results, the content of CH was evaluated as 28 wt%.

**Figure 5:**
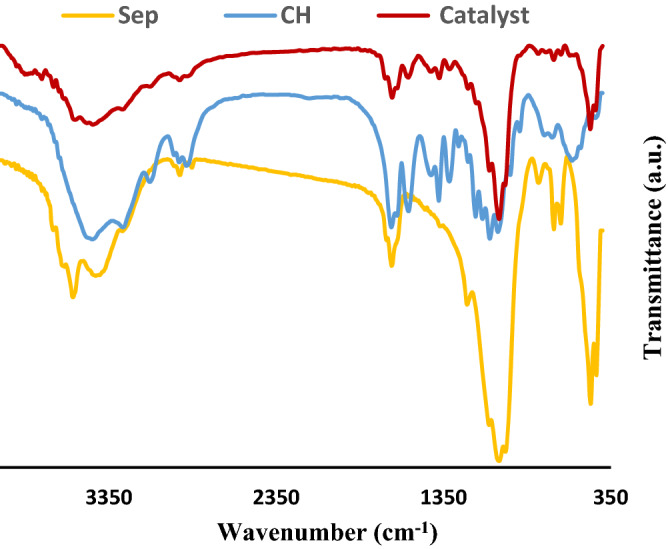
FTIR spectra of CH, Sep and Ag@Sep-N–CH.

To verify the structure of the inorganic–organic hybrid of Ag@Sep-N–CH, FTIR spectrum of Ag@Sep-N–CH was achieved and compared with that of each components, Fig. 5. In the spectrum of sepiolite, the absorbance band at 1,014 cm^−1^ is observed due to the Si–O stretching. Additionally, the absorbance bands discerned at 3,560, 3,428, and 645 cm^−1^ are representative of stretching and bending vibrations of –OH functionality^30^. In the FTIR spectrum of CH, the absorbance bands of CH at 3,444 cm^−1^, 1662 cm^−1^ can be observed. In the case of the FTIR spectrum of Ag@Sep-N–CH, all the discussed absorbance bands can be detected. However, as the distinctive bands of CH overlapped with that of sepiolite, FTIR spectroscopy cannot provide enough proofs for confirming the formation of the hybrid. Notably, the shifts of the bands can be attributed to the interactions between functionalized CH and sepiolite.

As the specific surface area is one of the most important features of catalysts, this value was estimated for Ag@Sep-N–CH via BET method. Moreover, the specific surface area of the catalyst was compared with that of sepiolite. The results approved that upon grafting CH, the specific surface area of sepiolite reduced from 161 to 130 m^2^ g^−1^. Moreover, upon introduction of silver nanoparticles, the pore volume and pore diameter reduced to 30.0 cm^3^ g^−1^ and 2.2 nm respectively. Furthermore, the recorded nitrogen adsorption–desorption isotherm was of the type II with H3 hysteresis loops, Figure S1^31^.

The XRD patterns of sepiolite and Ag@Sep-N–CH are illustrated in Figure S2. As shown the two XRD patterns are similar and displayed similar bands that can be assigned to sepiolite structure^8^. The reason for not observing the bands of silver nanoparticles can be accredited to low content of silver nanoparticles^32^.

**Figure 6:**
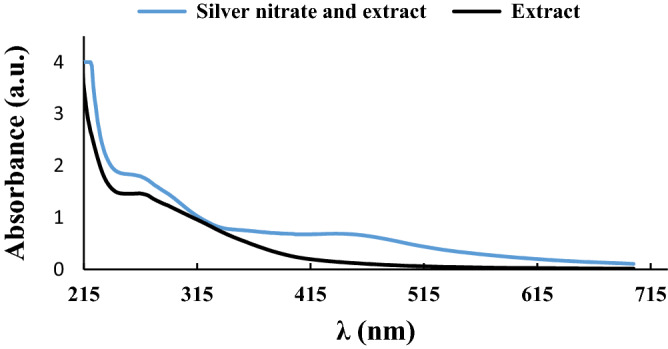
UV–visible spectra of Kombucha extract and the mixture of AgNO_3_ and Kombucha extract.

To verify the potential of the extract for reducing Ag(I) to Ag(0), UV–Vis spectra of Kombucha extract and the mixture of Kombucha extract and AgNO_3_ were obtained, Fig. 6. It is well-established that Ag(0) nanoparticles displayed a specific band in the UV–Vis spectrum at λ_max_ = 430 nm^33,34^ that can approve the reduction of Ag(I) to Ag(0). As shown, in Fig. 6, in the UV–Vis spectrum of the mixture of AgNO_3_ and Kombucha extract, the so-called characteristic band can be discerned, while this band is not appeared in the spectrum of Kombucha extract. This result indicated the successful reduction of silver salt via Kombucha extract.

The loading of silver nanoparticles on Sep-N–CH was measured by ICP analysis as 0.5 w/w%.

## Catalytic activity

To appraise the catalytic performance of Ag@Sep-N–CH, synthesis of xanthene derivatives from reaction of aldehydes and dimedone was targeted. In the onset, by using reaction of benzaldehyde and dimedone as a model reaction, the reaction parameters such as solvent, catalyst loading and temperature have been optimized. In this regard, first, the model xanthene derivative was synthesized by using 0.02 g Ag@Sep-N–CH in water as solvent at room temperature. It was revealed that (Table S1) in this condition only moderate yield of the desired product has been achieved. To improve the yield of the product, the reaction was examined in different solvents. It was determined that the best result was obtained in H_2_O: EtOH (1:2). Next, to elucidate the influence of the reaction temperature on the yield of the product, the model reaction was repeated at 50 and 70 °C. As listed in Table S1, the optimum reaction temperature was found as 50 °C. Finally, the influence of the loading of Ag@Sep-N–CH was scrutinized by using 0.02–0.04 g catalyst. The results demonstrated that increase of the catalyst loading from 0.02 to 0.03 g gave rise to the increase of the reaction yield. However, increase of this value to 0.04 g had no effect on the yield of the product.

After finding the optimum reaction condition, more precise study on the role of the hybrid components has been carried out. First, the necessity of silver nanoparticles on the structure of the catalyst was appraised. In this context, a control catalyst, Sep-N–CH was prepared similar to the reported procedure and its activity for catalyzing the synthesis of the model xanthene was examined. The result, Table 1, affirmed that Sep-N–CH exhibited moderate catalytic activity and led to the formation of the desired product in 50% yield. This observation confirmed the necessity of incorporation of silver nanoparticles for achieving high catalytic activity. Next, to elucidate the efficiency of CH-NH_2_ and Sep-Cl as catalyst supports and investigate whether there is any synergism between functionalized chitin and sepiolite, two samples, Ag@Sep-Cl and Ag@CH-NH_2_, were fabricated through wet impregnation of silver nanoparticles on CH-NH_2_ and Sep-Cl as supports and then the activities of these samples for the model reaction were measured. As shown in Table 1, these two samples displayed moderate catalytic activities that were inferior compared to Ag@Sep-N–CH. This observation confirms that hybrid of CH-NH_2_ and Sep-Cl is a more effective support compared to sole CH-NH_2_ and Sep-Cl. This can be due to the synergism between the hybrid components.

**Table 1:** Comparison of the catalytic activity of Ag@Sep-N–CH with some control catalysts.

Entry	Catalyst	Reaction time (h)	Yield (%)^a^
1	Ag@Sep-N–CH	3	95
2	Sep-N–CH	5	50
1	Ag@Sep-Cl	4	65
3	Ag@CH-NH_2_	3.5	78

^a^Reaction condition: 1 mmol of benzaldehyde and 2 mmol of dimedone and 0.03 g of catalyst at 50 °C in the mixture of H_2_O:EtOH (1:2).

In the next section, the scope of the present procedure is evaluated. In this regard, various aldehydes with electron-withdrawing and electron-donating groups were applied as starting materials for the synthesis of the corresponding xanthenes. Moreover, heterocyclic aldehyde, furfural has also been utilized as a substrate. The results, Table 2, established that all the used substrates could undergo the reaction using Ag@Sep-N–CH catalyst and led to the high yields of the corresponding xanthene derivatives. This result confirmed that this protocol can be generalized to diverse range of aldehydes.

**Table 2:** Synthesis of various xanthenes under Ag@Sep-N–CH catalysis.
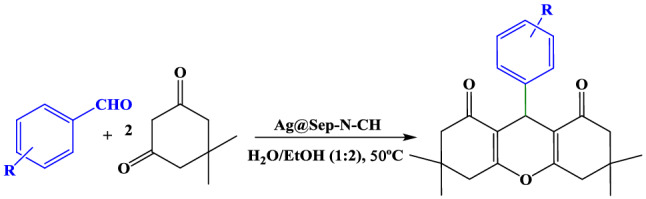

Entry	Substrate	Yield (%)
1	Benzaldehyde	95
2	4-NO_2_-benzaldehyde	90
3	2-NO_2_-benzaldehyde	98
4	4-Me-benzaldehyde	93
5	4-MeO-benzaldehyde	90
6	2-MeO-benzaldehyde	90
7	4-Cl-benzaldehyde	95
8	Furfural	90

## Comparison of the catalytic activity of Ag@Sep-N–CH with other catalysts

In the next part, Ag@Sep-N–CH performance for catalyzing the synthesis of the model xanthene was compared with some other catalysts, Table 3. Considering the fact that xanthenes are biologically-active compounds, various researchers focused on evolving efficient catalysts for their synthesis. Among the reported procedures in Table 3, some of them were not efficient and gave rise to low yields of the desired xanthene. Other tabulated catalysts showed competitive efficiency compared to Ag@Sep-N–CH. Although due to the different reaction condition, a precise comparison cannot be achieved, it can be inferred that Ag@Sep-N–CH can be recognized as an effective catalyst with comparable performance with reported catalysts. In detail, in the present protocol, high yield of the product can be achieved in aqueous media with use of relatively low catalyst loading at low temperature.

**Table 3:** The comparison of the performance of Ag@Sep-N–CH with some other catalysts.

Entry	Catalyst	Solvent	catalyst loading	Temp. (°C)	Yield (%)	References
1	Ag@Sep-N–CH	H_2_O:EtOH	0.03 g	50	95	This work
2	Nano-ZnO	–	10 mol%	100	Trace	^21^
3	Fe_2_(SO_4_)_3_.7H_2_O	–	10 mol%	120	86	^35^
4	Fe_3_O_4_@SiO_2_–SO_3_H	–	0.05 g	110	97	^22^
5	Silica-bonded S-sulfonic acid (SBSSA)	EtOH	0.03 g	Reflux	98	^23^
6	Nano-NiO	–	10 mol%	100	Trace	^21^
7	Barium Perchlorate	EtOH	15 mol%	Reflux	95	^36^
8	Nano titania-supported sulfonic acid (n-TSA)	–	0.013 g	90	91	^21^

## Reaction mechanism

**Figure 7:**
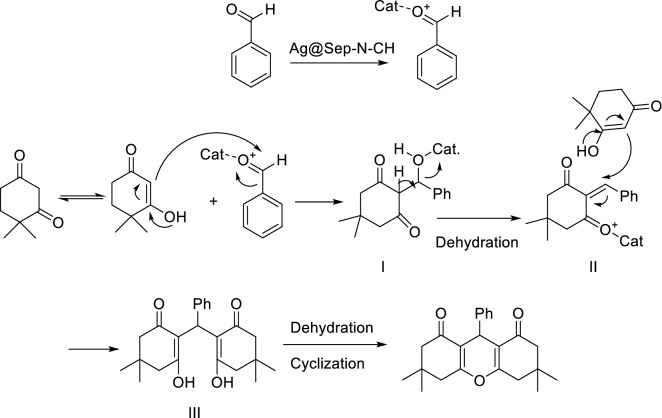
Plausible reaction mechanism for the synthesis of xanthenes.

According to the literature^37^, Ag@Sep-N–CH catalyzed synthesis of xanthene derivatives can be described as follow: Initially, Ag@Sep-N–CH activated the aldehyde. The activated aldehyde then reacted with the enol form of dimedone (Fig. 7) to generate an intermediate I. The latter underwent dehydration to form intermediate II. In the next step, intermediated II reacted with the second molecules of dimedone to furnish compound III. The latter then tolerated dehydration followed by cyclization to produce xanthene.

## Catalyst reusability

**Figure 8:**
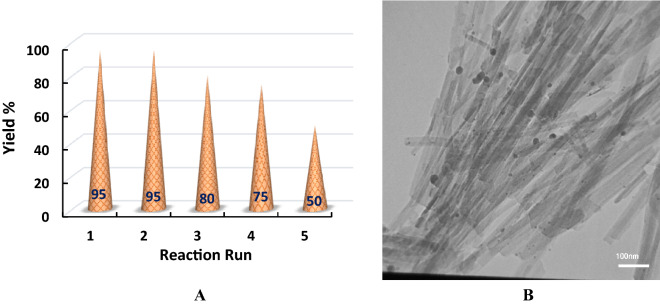
(**A**) Reuse of Ag@Sep-N–CH for the synthesis of the model xanthene derivative under optimum reaction condition. (**B**) TEM image of the reused Ag@Sep-N–CH after five runs of the model reaction under optimum reaction condition.

To examine the reusability of Ag@Sep-N–CH, the synthesis of the model xanthene derivative was carried out by using the reused Ag@Sep-N–CH under the optimum condition. It was found out that by using the reused catalyst, the yield of the model reaction did not alter and remained as 95%. Encouraged by this result, the reused catalyst was recovered and applied for the third run of the same reaction. The result, Fig. 8A, demonstrated that upon third use, the reaction yield decreased from 95 to 80%. Reuse for the fourth time also confirmed further decrement of the yield of the model xanthene (75%). Finally, use of Ag@Sep-N–CH for the fifth run of the reaction resulted in significant drop of the efficiency of the catalyst to 50%.

It was postulated that the reduction of the activity of Ag@Sep-N–CH can originate from leaching of silver nanoparticles that played the main role in the catalysis. To validate this assumption, Ag(0) loading of the recycled Ag@Sep-N–CH was measured via ICP method. It was determined that no leaching was occurred upon second run of the reaction. However, further reuse of Ag@Sep-N–CH triggered Ag(0) leaching and this value increased by increase of the number of cycles and reached to 10 wt.% of the initial loading of the fresh Ag@Sep-N–CH upon the fifth run.

The morphological change of Ag@Sep-N–CH after fifth run of the model reaction was also appraised. As shown in Fig. 8B, the TEM image of the reused Ag@Sep-N–CH affirmed that reuse of the catalyst caused agglomeration and increase of the average size of Ag(0) nanoparticles to some extent (the estimated mean particle size was 6.8 ± 1.5 nm). This issue can also contribute to the observed lower activity of the reused Ag@Sep-N–CH.

## Experimental

### Materials

Used chemicals for this research included, chitin (CH), Ag(NO_3_), (3‐amino propyl) triethoxysilane (APTES), (3-chloropropyl) trimethoxysilane (CPTES), dimedone, aldehydes, distilled water, toluene and ethanol. All chemicals were purchased from Sigma-Aldrich and used with no further purification. Sepiolite clay (Sep), PANGEL S9, was provided from TOLSA. The extract of that is a fermented mixture of yeast and bacteria was purchased from local store.

### Instrumentation and analysis

The applied instruments for the characterization of Ag@Sep-N–CH were follow: BRUKER, EQUINOX 55 apparatus for powder Fourier transform infrared (FT-IR) spectroscopy, METTLER TOLEDO instrument with heating rate of 10 °C min^−1^ for ), thermogravimetric analysis (TGA) under nitrogen atmosphere. Philips CM30300Kv instrument for transmission electron microscopy (TEM), BELSORP Mini II apparatus for Brunauer–Emmett–Teller (BET) analysis (degassing at 150 °C for 2 h). Siemens, D5000 apparatus using graphite monochromatic Cu-Kα for X-ray diffraction (XRD) analysis, UV–visible Spectrophotometer (PerkinElmer, Lambda 365) and Vista-pro for Inductively Coupled Plasma (ICP) analysis. Tescan instrument was used for recording SEM images.

## Catalyst fabrication

### Synthesis of the catalyst

#### Synthesis of Cl‐functionalized sepiolite (Sep‐Cl)

Sepiolite (2 g) was suspended in 80 mL toluene and mixed vigorously for 15 min. Subsequently, CPTES (2 mL) was added gradually and the mixture was sonicated for 30 min and then refluxed for 48 h at 110 °C under N_2_ atmosphere. At the end, the solid was filtered, washed several times with toluene and dried in a vacuum oven at 90 °C overnight.

#### Synthesis of amine‐functionalized chitin (CH-NH_2_)

A solution of APTES (2 mL) was added to a suspension of chitin (2 g) in dry toluene (40 mL). The mixture was then sonicated (power of 100 W) for 30 min and refluxed for 24 h at 110 °C. Afterwards, the resultant suspension was filtered and washed repeatedly with dry toluene and dried at 100 °C overnight.

#### Synthesis of sepiolite–chitin (Sep-N–CH)

Sepiolite-Cl (1.5 g) in dry toluene (30 mL) was suspended. Meanwhile, CH-NH_2_ (1.5 g) was crushed and well dispersed in toluene (30 mL) using ultrasonic irradiation of 100 W. Then, the two aforementioned suspension were mixed and refluxed for 24 h at 110 °C under N_2_ atmosphere. Upon completion of the reaction, the solid was filtered and placed in water. Then, the aqueous suspension was centrifuged. The obtained Sep-N–CH was gathered, washed with toluene repeatedly and dried in a vacuum oven at 100 °C.

#### Synthesis of Ag NPs and their embedding into Sep-N–CH: synthesis of Ag@Sep-N–CH

Sep-N–CH (1 g) was dispersed into a solution of AgNO_3_ (0.1 g) in deionized H_2_O (20 mL) under stirring condition at room temperature for 30 min. The silver ions were adsorbed onto the surfaces of Sep-N–CH via electrostatic attraction. Afterwards, the fresh extract of Kombucha (2 mL in 20 mL DW) as a reducing agent was added into the suspension. Upon addition of the Kombucha extract, the colour of the mixture darkened, which affirmed the reduction of silver ions to Ag(0). To stabilize Ag(0) NPs on Sep-N–CH, the mixture was stirred for 12 h. Finally, the product was separated and washed three times with EtOH/H_2_O and dried at 60 °C for 12 h, Fig. 1.

#### General procedure for xanthene synthesis

Aldehyde (1 mmol) and dimedone (2 mmol) were dissolved in 1:2 H_2_O: EtOH (3 mL). Then, Ag@Sep-N–CH (0.03 g) was introduced and the mixture was agitated at 50 °C for 3 h. At the end of the reaction, monitored by TLC, the reaction mixture was diluted with EtOH (15 mL) and Ag@Sep-N–CH was separated via simple filtration. Xanthene was then purified via recrystallization with EtOH. The yields of the reactions were measured by using GC.

#### Recycling of the catalyst

After the first run of the reaction, the catalyst was separated and washed with hot EtOH for three times. Subsequently, the recovered catalyst was dried in oven at 100 °C overnight and re-used for the next run of the reaction.

## Conclusion

With the aim of using naturally occurring compounds for the catalytic purposes, chitin and sepiolite have been functionalized and covalently grafted. Subsequently, the resulting hybrid, Sep-N–CH, was used as a support for the stabilization of silver nanoparticles, reduced by the extract of Kombucha. Interestingly, the resultant catalyst, Ag@Sep-N–CH, exhibited high catalytic activity for promoting one-pot reaction of dimedone and aldehydes in aqueous media to furnish xanthene derivatives in high yields. The study of some control samples, including Ag@Sep-Cl and Ag@CH-NH_2_ affirmed superior activity of Ag@Sep-N–CH. This result established that hybridization of CH-NH_2_ and Sep-Cl can improve the activity of the final catalyst due to the synergistic effects. The present protocol for xanthene synthesis showed broad substrate scope and various aldehydes could tolerate this reaction to furnish the corresponding products in high yields. The study of the recyclability of the catalyst confirmed that although Ag@Sep-N–CH maintained its activity for the second run, upon further reuse its activity dropped and reached from 95 to 50% after fifth run.

## Supplementary information

Supplementary Information.
