# Supplementary material for: Bio-assisted synthesized Ag(0) nanoparticles stabilized on hybrid of sepiolite and chitin: efficient catalytic system for xanthene synthesis
Source: Sci Rep. 2020 Sep 17;10:15285. doi: 10.1038/s41598-020-71866-2 (PMC7499264; doi:10.1038/s41598-020-71866-2)
Supplement: Supplementary file 1 — Supplementary Information. [file 41598_2020_71866_MOESM1_ESM.docx]

**Supporting information**

Bio-assisted synthesized Ag(0) nanoparticles stabilized on hybrid of sepiolite and chitin: Efficient catalytic system for xanthene synthesis

Fatemeh Ghoreyshi Kahangi^a^, Morteza mehrdad^a^, Majid M. Heravi^*b^, Samahe Sadjadi^*c^

**Figure S1.** A: The N_2_ adsorption-desorption isotherm of Ag@Sep-N-CH.

**Figure S2.** The XRD patterns of sepiolite and the catalyst.

Table S1. Optimization of the reaction condition for the synthesis of the model xanthene.

| Entry | Solvent | Temp. (ºC) | Catalyst Amount (g) | Yield (%) |
| --- | --- | --- | --- | --- |
| 1 | H_2_O | 25 | 0.02 | 60 |
| 2 | EtOH | 25 | 0.02 | 68 |
| 3 | H_2_O:EtOH (1:2) | 25 | 0.02 | 75 |
| 4 | THF | 25 | 0.02 | 55 |
| 5 | CH_3_CN | 25 | 0.02 | 50 |
| 6 | H_2_O:EtOH (1:2) | 50 | 0.02 | 80 |
| 7 | H_2_O:EtOH (1:2) | 70 | 0.02 | 90 |
| 8 | H_2_O:EtOH (1:2) | 50 | 0.03 | 95 |
| 9 | H_2_O:EtOH (1:2) | 50 | 0.04 | 95 |
